# Can ChatGPT pass the polish national medical specialization examination in orthopedics and traumatology?

**DOI:** 10.1007/s00402-026-06435-9

**Published:** 2026-08-10

**Authors:** Bartosz Maciąg, Krzysztof Bujak, Dawid Jegierski, Artur Stolarczyk

**Affiliations:** Department of Orthopedics and Rehabilitation, Medical University of Warsaw, Warsaw, Poland

**Keywords:** AI, ChatGPT, Exam, Orthopedics, Traumatology

## Abstract

**Introduction:**

Artificial intelligence (AI) has evolved rapidly in recent years and is becoming increasingly integrated into many areas of medicine. In the medical field, these systems have attracted considerable attention because of their potential applications in clinical decision support, medical education, scientific communication, and postgraduate training. The aim of the present study was to evaluate whether ChatGPT could achieve a passing score on the Polish National Medical Specialization Examination (Panstwowy Egzamin Specjalizacyjny, PES) in orthopedics and traumatology and to determine how different prompting strategies influenced its performance.

**Materials and methods:**

Authors systematically assessed the performance of ChatGPT-4 across five consecutive official PES exams (from Autumn 2023 to Spring 2025) in orthopedics and traumatology. Each exam was administered using three distinct prompting strategies: Professor prompt, Specialist prompt, Resident prompt. For each exam session (e.g., Spring 2024, Autumn 2023), the prompts were submitted to ChatGPT sequentially. The model’s responses were evaluated against the official answer key published by the Polish Center of Medical Exams (Centrum Egzaminów Medycznych, CEM).

**Results:**

The results were averaged across all prompts. The overall accuracy was 81% (range: 68.33%–85.83%). The highest score (85.83%) was recorded multiple times across different prompt types, indicating that the model could approach or exceed the minimum passing threshold depending on prompt structure. Agreement between prompting strategies was moderate to substantial (Cohen’s κ 0.518–0.632; Fleiss’ κ = 0.571). Radiology-related questions demonstrated the highest error rate among all question categories.

**Conclusions:**

Large language models such as ChatGPT can perform well on knowledge-based orthopedic examinations and may serve as useful tools for examination preparation and educational support. However, examination success should not be interpreted as evidence of clinical competence or readiness for independent surgical practice. Specialist certification and patient care remain dependent on human expertise, practical skills, and professional responsibility.

## Introduction

Artificial intelligence (AI) has evolved rapidly in recent years and is becoming increasingly integrated into many areas of medicine. Among its most prominent recent developments are large language models (LLMs), such as ChatGPT, which are capable of processing complex textual information and generating fluent, contextually appropriate responses. In the medical field, these systems have attracted considerable attention because of their potential applications in clinical decision support, medical education, scientific communication, and postgraduate training [1, 2]. Their ability to answer knowledge-based questions and provide seemingly reasoned explanations has raised growing interest in whether they can perform successfully in formal medical examinations [1, 3].

At the same time, the expanding use of LLMs in healthcare has generated important debate regarding accuracy, reliability, transparency, and accountability [2, 4]. Although these models can produce coherent and persuasive responses, such answers may not always be factually correct and may occasionally be misleading [2, 4]. These limitations are especially important in high-stakes settings, including certification and specialization examinations, where the accurate application of knowledge is essential. For this reason, standardized examinations may offer a useful and reproducible framework for evaluating both the potential and the limitations of LLMs in medically relevant tasks [3, 4].

Previous studies have shown that ChatGPT and related models can achieve notable results in a range of academic and professional examinations, including medical assessments [1, 3]. However, performance varies considerably depending on factors such as model version, examination language, specialty, question complexity, and prompting strategy [3, 5]. Despite these promising findings, most investigations have focused on English-language medical and orthopedic examinations, whereas evidence from national specialty certification examinations conducted in other languages remains limited [3, 7, 5]. Furthermore, the influence of different prompting strategies and multimodal examination content, including radiologic images, has not been adequately investigated in orthopedic specialty certification settings [17, 18].

The Polish National Medical Specialization Examination (Państwowy Egzamin Specjalizacyjny, PES) in Orthopedics and Traumatology provides a relevant framework for addressing these gaps. As a standardized national assessment administered within the legal framework governing postgraduate specialist training in Poland, it is intended to verify whether physicians completing specialty training have acquired the knowledge required for specialist certification [8, 9]. In addition, the examination includes both clinically oriented and image-based questions, allowing evaluation of ChatGPT performance in a non-English specialty certification setting.

The aim of the present study was to evaluate whether ChatGPT could achieve a passing score on the Polish National Medical Specialization Examination in Orthopedics and Traumatology, to assess the influence of different prompting strategies on model performance, and to examine performance consistency across five consecutive examination sessions.

## Materials and methods

### Study design and examination details

This retrospective observational study evaluated the performance of ChatGPT-4 on five consecutive official Polish Specialization Examination (Państwowy Egzamin Specjalizacyjny, PES) sessions in Orthopedics and Traumatology conducted between Autumn 2023 and Spring 2025. Each analyzed PES examination consisted of 120 single-best-answer multiple-choice questions with five response options (A-E). According to the examination regulations in force during the study period, candidates obtaining at least 75% of correct answers on the written examination were exempt from the oral component of the PES [8]. This threshold was used as the benchmark for determining whether ChatGPT achieved a passing examination result.

The official examination materials and answer keys published by the CEM were used as the reference standard. All questions from each examination session were included in the analysis.

### ChatGPT configuration and prompt definitions

Each exam was administered using three distinct prompting strategies:

- Professor prompt - structured and detailed prompt mimicking expert consultation.
- Specialist prompt - concise and focused prompt reflecting clinical reasoning.
- Resident prompt - more basic prompt representing general medical knowledge.

All examinations were performed using the ChatGPT platform (OpenAI, San Francisco, CA, USA) with the advanced reasoning model available at the time of testing. The analyses were conducted using the same model version throughout the study.

Each examination was uploaded as a complete original DOCX file containing all 120 questions together with the embedded radiographic images provided in the official examination materials. The examination files were processed as a whole rather than being entered question-by-question.

To minimize carryover effects and contextual memory, each examination session and each prompting strategy (Professor, Specialist, and Resident) were evaluated in separate newly initiated chat sessions. No custom instructions, external tools, web browsing functions, or additional contextual information beyond the examination materials and predefined prompts were provided. All responses were generated using the default settings of the ChatGPT platform available at the time of testing.

Three role-based prompting strategies were used. The Professor prompt instructed ChatGPT to respond as an experienced academic orthopedic surgeon involved in postgraduate education. The Specialist prompt instructed the model to respond as a board-certified orthopedic surgeon engaged in routine clinical practice. The Resident prompt instructed the model to respond as an orthopedic resident preparing for specialist certification. Apart from the assigned professional role, all prompts contained identical instructions regarding answer generation and response formatting.

For all prompting strategies, ChatGPT was instructed to select the single best answer for each question and provide responses in a standardized format suitable for subsequent comparison with the official examination answer key.

### Question classification and coding procedures

The questions were classified into *topic* and *type* categories. Incorrect responses were documented by item number, prompt type, and question.

Within the *topic* category, the following subgroups were defined:

- Arthroplasty - issues related to joint replacement procedures,
- Traumatology - topics concerning musculoskeletal trauma,
- Pediatric orthopedics (pediatric) - conditions specific to pediatric patients,
- Oncology - issues related to orthopedic oncology,
- General medical knowledge (general) - questions addressing general medical knowledge,
- Shoulder - questions related to the glenohumeral joint not classified under other thematic groups,
- Hand - questions concerning the hand and elbow region not classified under other thematic groups,
- Hip - questions specific to the hip joint not classified under other thematic groups,
- Knee - questions specific to the knee joint not classified under other thematic groups,
- Foot and ankle (foot) - questions related to the foot and lower leg region not classified under other thematic groups,
- Spine - questions related to the vertebrae not classified under other thematic groups.

Within the *type* category, questions were classified as follows:

- Radiology - questions including radiographic imaging (X-ray, CT, or MRI) and directly referring to it in the clinical context,
- Case-based - questions presenting a clinical scenario requiring diagnostic or therapeutic decision-making,
- Combination - questions in which the correct answer consisted of a combination of the proposed options (e.g., 1,2,3,4; 2,3,5; 4,5),
- Standard multiple-choice (standard) - conventional single-best-answer questions with a typical set of response options.

Importantly, all 120 questions in the examination were single-best-answer multiple-choice questions. The term “combination” refers only to the structure of the answer options and does not indicate that candidates could select more than one answer.

### Image handling protocol

ChatGPT-4 received the original examination files in DOCX format. All radiographic images, including X-rays, CT scans, and MRI scans, were embedded within the documents directly below their corresponding questions, reproducing the layout of the official examination materials.

No additional image descriptions, annotations, or investigator-generated interpretations were provided. Consequently, radiology questions were evaluated using both the textual content and the original visual materials available within the examination files.

### Statistical analysis methods

Descriptive statistics were used to summarize examination performance across prompting strategies. Results are presented as percentages, counts, and proportions.

Agreement between prompting strategies was assessed on a question-by-question basis using Cohen’s kappa coefficient for pairwise comparisons between Professor, Specialist, and Resident prompts. Overall agreement among all three prompting strategies was evaluated using Fleiss’ kappa coefficient.

Kappa values were interpreted according to the criteria proposed by Landis and Koch, where values below 0.20 indicate slight agreement, 0.21–0.40 fair agreement, 0.41–0.60 moderate agreement, 0.61–0.80 substantial agreement, and values above 0.80 almost perfect agreement.

Differences in response patterns between prompting strategies were evaluated using McNemar’s test for paired binary data. A p-value below 0.05 was considered statistically significant.

### Quality assurance

To ensure reproducibility and consistency, all examination materials were obtained from official CEM publications and processed without modification. The same examination files were used across all prompting strategies. Response scoring was performed against the official answer keys, and classification procedures were conducted according to predefined criteria.

Data extraction, coding, and result verification were independently reviewed by the investigators. Any discrepancies identified during the review process were resolved through consensus prior to statistical analysis.

## Results

**Chart 1:**
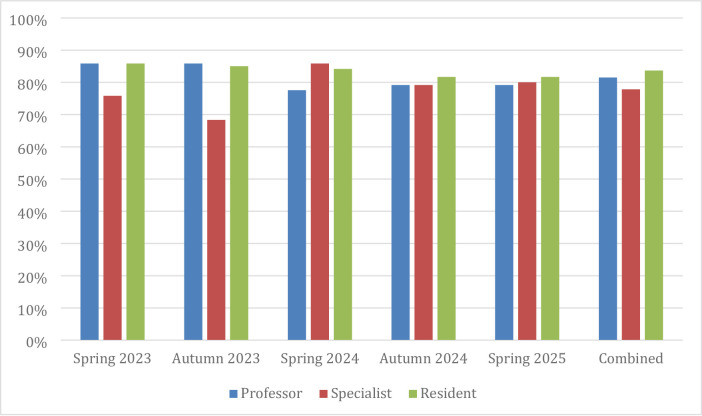
Chat GPT performance on PES exams by prompt type

The summarized results from the five examination sessions are presented in Chart 1. When the results were averaged across all prompts, the overall accuracy was 81% of correct responses (range: 68.33%-85.83%). For comparison, the 439 examinees who took the examination during the analyzed years achieved a mean score of 57.64%.

Resident-style prompts demonstrated the most consistent performance across examination sessions and achieved the highest score in several of them. The highest score (85.83%) was recorded multiple times across different prompt types, indicating that the model frequently exceeded the examination passing threshold.

Agreement analysis demonstrated moderate to substantial concordance between prompting strategies. The highest agreement was observed between the Professor and Resident prompts (Cohen’s κ = 0.632), whereas agreement between the Specialist prompt and the remaining strategies was lower. Overall agreement among all three prompting approaches was moderate (Fleiss’ κ = 0.571). McNemar testing demonstrated significant differences in response patterns between the Specialist prompt and both the Professor (*p* = 0.016) and Resident prompts (*p* < 0.001), whereas no significant difference was observed between the Professor and Resident prompts (*p* = 0.208).

**Chart 2:**
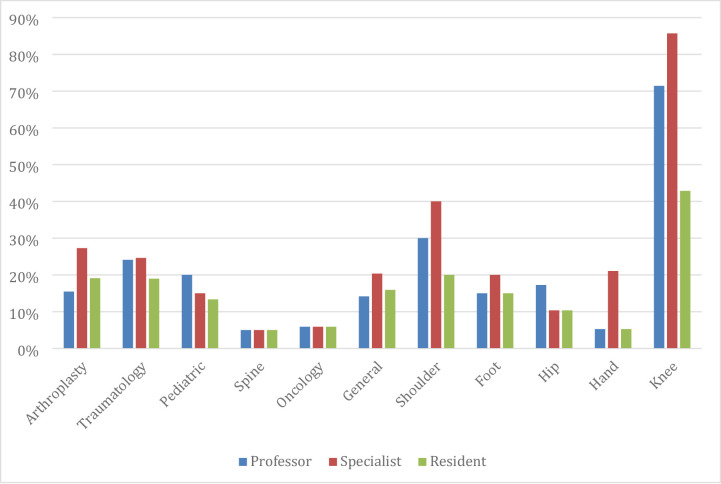
Errors committed by individual prompts (topic)

**Chart 3:**
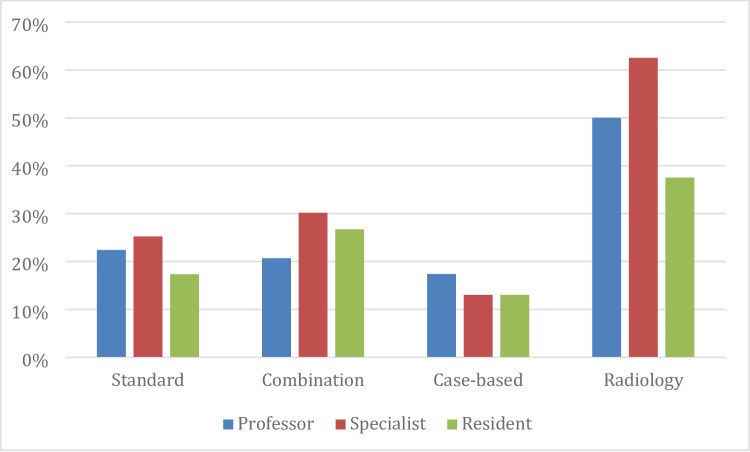
Errors committed by individual prompts (type)

The distribution of errors across individual prompts is presented in Charts 2 and 3. Mean error rates across all prompting strategies are presented in Tables 1 and 2. Analysis of these data indicates that the model exhibited similar error patterns across prompting strategies. The highest error rates were observed for questions related to the knee and shoulder, with mean incorrect response rates of 67% and 30%, respectively. Regarding question type, radiology-related questions demonstrated the poorest performance, with an average error rate of 38%.

**Table 1:** Errors committed by all the prompts (topic)

Topic	Number	Percent
Arthroplasty	68	20,61%
Traumatology	132	22,56%
Pediatric	29	16,11%
Spine	3	5,00%
Oncology	3	5,88%
General	57	16,81%
Shoulder	9	30,00%
Foot	10	16,67%
Hip	11	12,64%
Hand	6	10,53%
Knee	14	66,67%

**Table 2:** Errors committed by all the prompts (type)

Type	Number	Percent
Standard	206	21,66%
Combination	90	25,86%
Case-based	10	14,49%
Radiology	36	50,00%

Across all examinations, the distribution of questions by topic demonstrated that the highest proportion concerned traumatology, general medical knowledge, and arthroplasty (Table 3). Regarding question type, standard multiple-choice questions were by far the most prevalent format (Table 4).

**Table 3:** The distribution of questions by topic across all examinations

Topic	Number	Percent
Arthroplasty	110	18,33%
Traumatology	195	32,50%
Pediatric	60	10,00%
Spine	20	3,33%
Oncology	17	2,83%
General	113	18,83%
Shoulder	10	1,67%
Foot	20	3,33%
Hip	29	4,83%
Hand	19	3,17%
Knee	7	1,17%

**Table 4:** The distribution of questions by type across all examinations

Type	Number	Percent
Standard	395	65.83%
Combination	116	19,33%
Case-based	23	3,83%
Radiology	24	4,00%

## Discussion

The findings of the present study suggest that, under selected prompting conditions, ChatGPT- can consistently exceed the formal passing threshold of the Polish national specialty examination in orthopedics and traumatology. This observation is broadly consistent with previous studies showing that large language models can perform competitively in medical knowledge assessments, including licensing and specialty-oriented examinations. GPT-4 has been reported to exceed the passing threshold on USMLE-style material, while earlier work also demonstrated that ChatGPT could achieve performance within the passing range on parts of the USMLE. More recent evidence from systematic reviews indicates that model performance in medical examinations varies substantially across model versions, languages, specialties, and study designs, but that newer-generation models generally outperform their predecessors [10–12].

Our findings are also in line with prior research in orthopedics. Previous studies evaluating ChatGPT and GPT-4 on the Orthopaedic In-Training Examination (OITE) and other board-style orthopedic question sets demonstrated that these models can achieve moderate-to-strong results, in some cases approaching the level of orthopedic trainees. At the same time, performance has not been uniform across studies, which suggests that examination structure, question style, exclusion of image-based items, and model version may substantially influence final scores [6, 13–16].

An important aspect of the present study is that it extends existing evidence beyond the predominantly English-language examinations evaluated in previous reports [1, 3, 5]. The PES represents a national specialty certification examination conducted within a different linguistic, educational, and regulatory environment than those typically assessed in the literature [8, 9]. Despite these differences, ChatGPT demonstrated performance comparable to that reported in studies based on USMLE- and OITE-style examinations [5, 13–16]. This observation suggests that the model’s ability to retrieve and apply orthopedic knowledge may be relatively robust across examination systems and languages, although further studies are needed to determine the extent to which language-specific factors influence performance [3, 5].

An additional finding of interest was the consistently strong performance observed with the Resident prompt, which in several examination sessions slightly outperformed both the Professor and Specialist prompts. The Specialist prompt demonstrated significantly different response patterns compared with both the Professor and Resident prompts. Examination of the available error distribution showed that the excess errors were not restricted to one subject area or to structurally complex questions. Notably, the largest absolute difference between the Specialist and Resident prompts occurred in standard single-best-answer items. This finding does not support a simple explanation based solely on question complexity or over-analysis.

Because structured reasoning narratives and calibrated confidence estimates were not prospectively collected, the internal basis of these differences cannot be determined from the present data. Assigning mechanisms such as over-complication, false confidence, or constraint-induced confusion would therefore be speculative. The findings instead indicate that role-based prompting may influence answer selection in a non-linear and potentially unpredictable manner.

This observation is consistent with previous reports suggesting that more complex expert-oriented prompts do not necessarily improve performance and may occasionally introduce unnecessary cognitive constraints for the model [21, 22]. In contrast, simpler prompting strategies may facilitate more direct retrieval of relevant information and better align with the distribution of educational and examination-style content encountered during model training. The comparatively lower performance of the Specialist prompt further suggests that prompt structure may influence model outputs in ways that are not intuitively related to the level of expertise being simulated. Future studies should investigate these relationships systematically.

Agreement analysis provided additional insight into the relationship between prompting strategies. Although Cohen’s kappa coefficients demonstrated moderate-to-substantial agreement between prompts and Fleiss’ kappa indicated moderate overall agreement, McNemar testing revealed significant differences in response patterns involving the Specialist prompt. These findings suggest that prompt formulation influenced not only overall examination scores but also the specific questions answered correctly or incorrectly. Consequently, prompt engineering may affect the way knowledge is retrieved and applied even when final performance appears broadly similar.

An analysis of the error distribution in our study showed a relatively high proportion of incorrect answers in radiology-related questions. Unlike several previous orthopedic examination studies that either excluded image-based questions or evaluated them without providing the original images to the model, the present analysis retained the original examination format, including embedded radiographic images [13–15]. Consequently, the observed performance may more closely reflect real-world specialty examination conditions and provide additional insight into the capabilities and limitations of multimodal large language models in orthopedic assessment settings [17, 18]. This finding is clinically plausible. Although AI systems have shown promising results in selected imaging tasks, particularly fracture detection and pattern recognition on radiographs, current multimodal large language models remain limited in the interpretation of complex medical imaging and in the integration of imaging findings with broader clinical context, even when image data are available. Multimodal systems continue to face important challenges related to image understanding, hallucinated interpretations, limited transparency, and variable generalizability across real-world settings [17–20]. In the context of a specialty examination, where questions often require the simultaneous interpretation of imaging findings, anatomy, mechanism of injury, and therapeutic implications, such limitations may substantially contribute to model error.

With regard to anatomical subcategories, a relatively high proportion of incorrect responses was observed in questions related to the knee and shoulder. However, this result should be interpreted cautiously. These categories accounted for only a small fraction of the total question pool, and the apparent increase in error rate may therefore reflect sampling instability rather than a true deficit in model performance within these domains. In larger content categories, performance appeared more stable, which supports the possibility that at least part of the observed variability was driven by the limited number of items rather than by consistent topic-specific weakness.

Several broader limitations of LLM-based performance in specialist examinations should also be emphasized. First, success in multiple-choice testing should not be equated with genuine clinical competence. LLMs generate probabilistic text outputs on the basis of patterns in training data and do not possess embodied clinical judgment, situational awareness, or professional accountability. Second, model outputs may be sensitive to prompt wording and response format, which introduces a degree of variability that is difficult to reconcile with the consistency required in high-stakes clinical decision-making. This issue is particularly relevant in studies such as ours, in which prompting strategy itself constitutes an experimental variable [11, 12, 21, 22].

These concerns may be especially important in orthopedics and traumatology. Specialist practice in this field extends far beyond declarative knowledge and includes manual skills, intraoperative adaptability, tactile feedback, team communication, and time-sensitive decision-making under uncertainty. Even highly capable language models cannot perform physical examination maneuvers, assess tissue quality, manipulate instruments, control bleeding, or modify surgical strategy in response to intraoperative findings. Therefore, although LLMs may demonstrate substantial utility in educational support, examination preparation, and structured knowledge retrieval, they cannot substitute for the experiential, procedural, and ethical dimensions of specialist medical practice [16, 18, 19].

Taken together, the present findings indicate that ChatGPT may be capable of answering a substantial proportion of specialist examination questions correctly, and under favorable prompting conditions may approach formal passing thresholds. However, this should not be interpreted as evidence of readiness for autonomous clinical practice. Rather, the results support the view that LLMs may serve as adjunctive tools in medical education and knowledge support, while responsibility for diagnosis, treatment decisions, and procedural care must remain with trained physicians.

## Conclusion

This study supports the idea that AI language models like ChatGPT can perform well on knowledge-based medical exams. These findings suggest that large language models may have a valuable role in medical education and examination preparation.

However, examination success should not be interpreted as evidence of clinical competence or readiness for independent practice. Even if an AI model answers 90% of questions correctly, it does not mean it can safely manage patients, build therapeutic relationships, or take responsibility for outcomes. It certainly cannot perform surgery.

AI can and should assist in medical education, especially for test preparation or knowledge reinforcement. Yet, it remains a tool - not a replacement. In orthopedics, where physical skill and human judgment are central, the role of AI must be complementary, not competitive.

## Data Availability

All data supporting the findings of this study are available within the paper and its Supplementary Information.
